# 

**Published:** 1902-11-15

**Authors:** 


### CONTENTS.

Nitrous Oxid Gas, - - - *By S. Straith, D.D.S.* 541
Irregularities and How to Correct Some of Them, *By J. H. Stofflett, D.D.S.* 553
Importance of the Conservation of the First Permanent Molar, - - *By A. C. Runyan, D.D.S.* 560
Mechanical Devices for Cleft Palate, *By Dr. H. J. Jaulusz* 565
What Art has Done for Dentistry—and what Dentistry has Done for Art, - - *By Dr. B. J. Cigrand* 569
How to Construct an Upper Vulcanite Denture Quickly, *By C. H. Warboys, D.D.S.* 571
Somnoforme; A New Anesthetic, - *By W. H. T.* 573
The Use of Adrenalin as an Addition to Solutions for Local Anesthesia, *By Charles A. Elsberg, M.D.* 576
The British Dental Association and the National Dental Association of the United States Contrasted, *By William H. Trueman, D.D.S.* 578
Judge Scores Dental School. Refuses Mandamus for License to a German-American College Graduate 582
Use of Calcium Chlorid to Prevent Hemorrhage After Tooth Extraction - - - - - - - 583
Plastic Fillings of Metallic Salts, *By Prof. Ernest Schmitt* 584
Entrance Requirements to German Dental Schools - 585

